# Prone positioning during venovenous extracorporeal membrane oxygenation for acute respiratory distress syndrome: a systematic review and meta-analysis

**DOI:** 10.1186/s13054-021-03723-1

**Published:** 2021-08-12

**Authors:** Wynne Hsing Poon, Kollengode Ramanathan, Ryan Ruiyang Ling, Isabelle Xiaorui Yang, Chuen Seng Tan, Matthieu Schmidt, Kiran Shekar

**Affiliations:** 1 Yong Loo Lin School of Medicine, National University of Singapore, Singapore, Singapore; 2 Cardiothoracic Intensive Care Unit, National University Heart Centre, National University Hospital, Level 9, 1E Kent Ridge Road, Singapore, 119228 Singapore; 3 Saw Swee Hock School of Public Health, National University of Singapore, Singapore, Singapore; 4 Service de Médecine Intensive-Réanimation, Institut de Cardiologie, Assistance Publique-Hôpitaux de Paris, Hôpital Pitié-Salpêtrière, Paris, France; 5 Sorbonne Université, GRC 30, Reanimation et Soins Intensifs du Patient en Insuffisance Respiratoire aigüE, AP-HP, Hôpital de la Pitié Salpêtrière, 75013 Paris, France; 6 Adult Intensive Care Services, Prince Charles Hospital, Brisbane, QLD Australia; 7 Queensland University of Technology, Brisbane, Australia; 8 University of Queensland, Brisbane, Australia; 9 Bond University, Gold Coast, QLD Australia

**Keywords:** Prone positioning, Extracorporeal membrane oxygenation, Adult, Acute respiratory distress syndrome

## Abstract

**Background:**

Prone positioning (PP) improves oxygenation and respiratory mechanics and is associated with lower mortality in patients with moderate to severe acute respiratory distress syndrome (ARDS). Despite this, some patients develop refractory hypoxemia and hypercapnia requiring venovenous extracorporeal membrane oxygenation (VV ECMO) support and are usually cared for in supine position. The physiologic and outcome benefits of routine PP of patients during VV ECMO remains unclear. Hence, we conducted the systematic review and meta-analysis to evaluate the outcome benefits of PP for patients with ARDS being treated with VV ECMO.

**Methods:**

After registration with PROSPERO (CRD42020199723), MEDLINE, EMBASE, Scopus and Cochrane databases were searched for relevant studies that reported PP in more than 10 adult patients supported with VV ECMO from origin to 1 March 2021. Studies were reviewed for quality using appropriate Joanna Briggs Institute (JBI) checklists, and certainty of evidence was assessed using the GRADE approach. The random-effects model (DerSimonian and Laird) was used. The primary outcome of interest was cumulative survival. Secondary outcomes were intensive care unit length of stay (ICU LOS) and ECMO duration. Changes in arterial blood gas (ABG) values, ventilator mechanics and complication rates were also studied.

**Results:**

Of 812 potentially relevant publications, 12 studies (640 patients) met our inclusion criteria. Due to overlapping study populations, 11 studies were included in the final meta-analysis. Cumulative survival in patients that underwent PP was 57% (95% CI 41.9–71.4, high certainty). Patients that underwent PP had longer ICU LOS (+ 14.5 days, 95% CI 3.4–25.7, *p* = 0.01) and ECMO duration (+ 9.6 days, 95% CI 5.5–13.7, *p* < 0.0001). After PP, patients had significantly higher PaO_2_/FiO_2_ ratio, lower PaCO_2_ and reduced ventilator driving pressure, and no major complications were reported.

**Conclusions:**

PP during VV ECMO appears safe with a cumulative survival of 57% and may result in longer ECMO runs and ICU LOS. However, evidence from appropriately designed randomized trials is needed prior to widespread adoption of PP on VV ECMO.

**Supplementary Information:**

The online version contains supplementary material available at 10.1186/s13054-021-03723-1.

## Background

Prone positioning (PP) has been shown to improve outcomes in patients with acute respiratory distress syndrome (ARDS). The PROSEVA trial reported significantly lower 28- and 90-day mortality in patients that underwent PP, when compared with supine positioning in patients with ARDS [1]. It has also been postulated that survival benefits of prone ventilation are independent of improvement in gas exchange and that it results from the ability of PP to reduce ventilator induced lung injury (VILI). Multiple mechanisms have been proposed to account for this effect, including reduction in alveolar overinflation and equalization of pleural pressure, leading to parenchymal homogeneity and better oxygenation [1–3]. PP relieves lung stress and improves respiratory mechanics by offloading the pressure of the heart and abdominal contents on the diaphragm leading to enhanced clearance of pulmonary secretions and reduced lung injury [4].

The role of venovenous extracorporeal membrane oxygenation (VV ECMO) in the management of ARDS has changed from being a rescue therapy to a more definitive therapy in the recent years [5]. It has a more decisive role in the management of hypoxemia that is refractory to standard management that includes low tidal volume ventilation, paralytic agents and PP [6–8]. While both VV ECMO and PP have independently been shown to improve patient outcomes in ARDS, the combination of both therapies has not been studied in great detail. The risks and benefits of enhancing lung recruitment during refractory hypoxemia on VV ECMO is still unclear [9]. Equally, it is not fully clear to what extent PP improves pulmonary mechanics and right ventricular function and whether these improvements result in meaningful clinical benefits. Additionally, the limited literature available on the use of PP during VV ECMO has suggested great variability in patient selection and outcomes, with some depicting improvement in PaO_2_/FiO_2_ ratios but highlighting failure to improve respiratory compliance as well as increased ECMO-related complications [10, 11]. We conducted a systematic review and meta-analysis on the use of PP during ECMO for adult ARDS aiming to gain a better understanding of the inter-therapeutic relationship and evaluate patient survival on this regimen.

## Methods

### Search strategy and study selection

The protocol for this systematic review and meta-analysis is registered with PROSPERO (CRD42020199723). A systematic search was conducted following the Preferred Reporting Items for Systematic Reviews and Meta-Analysis (PRISMA) Statement, including the following keywords and their variations: “Extracorporeal Membrane Oxygenation” and “Prone Positioning” from origin to 1 March 2021 and all relevant studies and their citation lists were assessed for inclusion (Additional file 1: Table S1).

Studies were included if they were written in English and included adult (≥ 18 years) patients undergoing ECMO for ARDS in which PP was explicitly described, and the outcomes of PP therapy such as patient survival were clearly indicated. The primary outcome was cumulative survival based on the longest interval survival reported, and secondary outcomes were mean differences in intensive care unit length of stay (ICULOS) and ECMO duration, changes in arterial blood gas (ABG) and ventilatory parameters, as well as incidence of complications. Inclusion of articles was not limited by type of study or publication year, but reviews of Extracorporeal Life Support Organization (ELSO) registry data were excluded to avoid duplication of reported patients. In studies taking place at the same institution across overlapping time periods, the study with the larger number of patients was included, and all others were excluded. Exclusion criteria included studies reporting on less than 10 patients or non-human studies, review articles and case reports. The eligibility of studies was assessed by going through the title and abstracts. Full texts of the shortlisted articles were then searched and evaluated for inclusion. Authors were contacted for additional data, if required.

### Data extraction

Data extraction were conducted independently using a prespecified datasheet. Extracted data included study characteristics (study duration, design, year of publication, country of study center), patient demographics (number, gender, age, comorbidities, BMI), clinical baseline parameters (ARDS etiology (direct (primary, or pulmonary) ARDS and indirect (secondary, extrapulmonary) ARDS), baseline PaO_2_/FiO_2_), ECMO and PP details (duration between ECMO implantation to PP, PP indications, duration of PP, number of PP sessions, as well as the type of PP regime used [fixed duration or variable durations], control details). Outcome data on patient survival (to ECMO weaning, to ICU discharge, to hospital discharge) as well as data on ECMO duration, ICU LOS, ABG and ventilator parameters pre-PP and post-PP (after completion of at least one PP cycle) as well as any complications reported were collected.

### Assessment of risk of bias and certainty of evidence

Risk of bias was independently assessed using the appropriate Joanna Briggs Institute (JBI) Critical Appraisal Checklists. Egger’s test was also used to evaluate for publication bias. We used the Grading of Recommendations, Assessments, Developments and Evaluations (GRADE) approach to assess the certainty of evidence. (GRADEpro app available online: https://www.gradepro.org [accessed on 4 March 2021)].

Screening of articles for inclusion, data extraction, and risk of bias assessment were conducted independently by three authors (WHP, RRL, IXY). Any conflicts were resolved by consensus or appeal to a fourth reviewer (KR).

### Statistical analysis

Statistical analyses were performed using R 3.6.2, using *meta* and *dmetar* packages [12]. Pooling of means and standard deviations from aggregate data presented in each study was conducted as per Wan et al. [13] Significant interstudy heterogeneity was expected given the variability of the ICU patients and response to intervention. As such, random-effects meta-analyses (DerSimonian and Laird) were conducted using the Freeman-Tukey double arcsine transformation, and 95% confidence intervals (CIs) were computed using the Clopper-Pearson method [14–16]. In addition, due to the heterogeneity of reported data, cumulative survival—pooling survival proportions from longest post-discharge timepoint reported—was conducted for a better overall picture of the data available. Where available, propensity-score matched and/or risk-adjusted data were used in meta-analysis of primary and secondary outcomes. Survival outcomes are presented as pooled proportions, binary outcomes were presented as pooled relative risks (RRs), and continuous outcomes are presented as pooled means or standardized mean differences, all with corresponding 95% CIs.

### Subgroup/sensitivity analysis

Planned subgroup analyses included influence of study population (Coronavirus Disease 2019 (COVID-19) vs. Non-COVID-19), type of PP regimen, duration to PP initiation, ECMO duration and JBI score. Analyses of RRs for cumulative mortality and complication incidence were conducted with continuity correction to allow for the inclusion of studies with zero events [17]. Sensitivity analysis was conducted by excluding studies with JBI score < 8 in the primary meta-analysis. Summary level metaregression was conducted to explore potential sources of heterogeneity or prognostically relevant study-level covariates. Planned metaregression analyses included influence of age, gender, ARDS etiology, baseline PaO_2_/FiO_2_ (PF) ratio, JBI score and year of publication.

## Results

### Study details and demographics

Of 812 potentially relevant publications, 27 full text publications were reviewed. Twelve observational studies of 640 patients met our inclusion criteria [10, 17–27] Of the 12 studies, 11 were retrospective and one was prospective in nature; three were multi-center studies and the remaining were single-center studies. There was an overlap between two of the included studies [20, 21], eleven studies (625 patients) were included in the meta-analysis [10, 17–19, 21–27] (Additional file 1: Fig. S1). Six studies were two-armed comparative studies, and the remaining were single arm observational studies. Three studies provided data on propensity-score matched or risk adjusted cohorts, which were used in analysis of primary and secondary outcomes. In total, there were 363 patients who underwent PP and 262 control patients in this pooled matched cohort. There were variations in ARDS definitions across included studies: seven studies used the Berlin definition; one study used the Murray Lung Injury Score (LIS), and three studies did not report a definition of ARDS. Ten studies reported the etiology of ARDS amongst studied populations. Of these, a larger proportion of patients suffered from ARDS due to direct lung injury 87% (95% CI 69.3–98.6) as opposed to ARDS from secondary injury. Three studies of 181 patients described the use of ECMO and PP for ARDS in the setting of COVID-19 infection.

Key characteristics of the included studies are summarized in Additional file 1: Table S2. Briefly, the pooled mean age of all patients was 50 years (95% CI 47.0–53.7), pooled mean BMI was 30 (95% CI 28.1–31.9), and the pooled proportion of males was 70% (95% CI 65.4–74.6). There were no significant differences in patient demographics between the prone and control groups. Pre-PP ECMO duration was reported in 7 studies, and the pooled duration was found to be 4.9 days (95% CI 2.5–7.3).

### Assessment of study quality

Appraisal of studies using the JBI checklists for prevalence studies suggested high level of quality across the studies included for this review, as majority of the studies were scored 8 or higher out of a maximum of 9 points (Additional file 1: Table S3). Egger’s test yielded nonsignificant results for publication bias. An evidence table of our GRADE assessments is presented in Additional file 1: Tables S4 and S5.

### Primary outcome

The primary outcome of interest was pooled cumulative survival. Included studies reported varying survival interval data, including survival to ICU discharge, hospital discharge as well as 30-day, 60-day and 90-day survival.

**Fig. 1:**
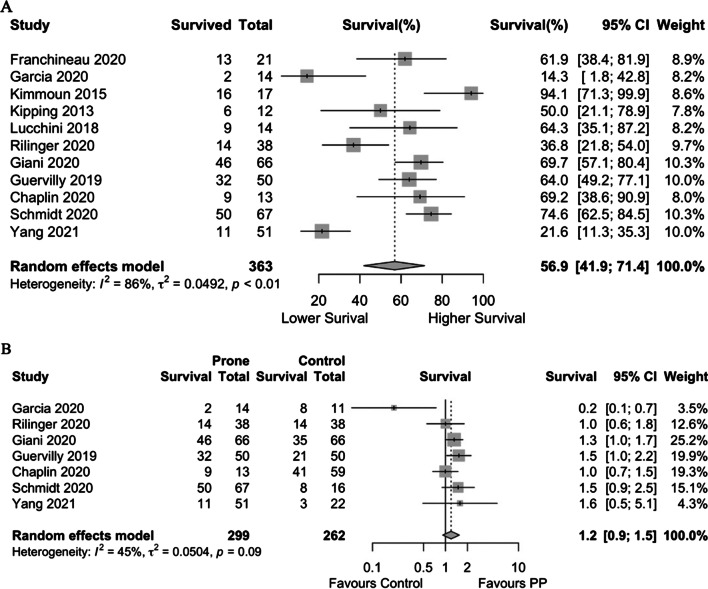
Forest plot for pooled cumulative survival of patients who underwent prone positioning (**a**) and forest plot for comparison of cumulative survival in proned and control group patients (**b**)

The cumulative survival (pooling survival proportions from longest post-discharge timepoint reported) in patients that underwent PP (11 studies, 363 patients) was 57% (95% CI 41.9–71.4, high certainty, Fig. 1a). Pooled proportion of survival to hospital discharge in patients that underwent PP (7 studies, 211 patients) was 58% (95% CI 37.6–77.9); 4 studies reported on RRs for survival. Patients who underwent PP had a higher non-significant chance of survival to discharge (4 studies, RR = 1.18, 95% CI 0.96–1.46, *p* = 0.11). Pooled survival to 30-days post-discharge was 50% (3 studies, 95% CI 21.4–79.1), reported 60-day survival of 72% (2 studies, 95% CI 63.6–80.5), 1 study reported 90-day survival of 64% for patients that underwent PP and 42% for the control group. Cumulative survival amongst patients who underwent PP compared to those who did not, was nonsignificant (7 studies, RR: 1.19, 95% CI 0.92–1.55, *p* = 0.19, Fig. 1b).

Sensitivity analysis for the primary outcome of pooled cumulative survival was conducted by leaving out studies with JBI score < 8. After sensitivity analysis, the pooled cumulative survival for patients undergoing PP was 56% (8 studies, 95% CI 36.9–73.9), and the chances for cumulative survival was 1.23 (6 studies, 95% CI 0.90–1.68, *p* = 0.19).

### Metaregression and subgroup analysis

Subgroup analyses based on the inclusion of patients with COVID-19, ECMO to PP interval, total ECMO duration, type of PP regime and quality of studies by JBI scoring were conducted. These analyses were conducted to elicit possible sources of variability in the primary outcome between groups, such as differing ARDS phenotypes between COVID-19 and non-COVID-19 patients. There was significant difference in the cumulative survival of patients with longer ECMO duration (more than 14 days) than patients with shorter ECMO durations (Table 1).

**Table 1:** Results of subgroup analysis for pooled cumulative survival among patients who underwent prone positioning

Subgroup	No. of studies	Pooled proportion survived/%	95% CI	*p* value for interaction
Study population				
Non-COVID-19	8	64	51.7–75.9	0.24
COVID-19	3	37	4.0–78.7	
Proning initiation				
< 5D ECMO	3	55	27.4–81.2	0.21
> 5D ECMO	3	78	55.1–95.1	
ECMO duration				
< 14D	3	33	16.3–51.9	0.0002
> 14D	7	71	63.8–78.5	
Proning regimen				
Fixed duration	4	73	55.4–87.5	0.12
"As tolerated"	6	54	36.9–70.0	
JBI score				
100%	6	61	40.3–79.5	0.57
< 100%	5	52	30.4–73.4	

*95% CI* 95% confidence interval, *COVID-19* Coronavirus Disease 2019, *ECMO* extracorporeal membrane oxygenation, *JBI* Joanna Brigg’s Institute

Covariates included in the univariate metaregression analysis of cumulative survival were mean age of patients, proportion of males in sample, etiology of ARDS, pre-ECMO PF ratio, pO2, pH, study quality as measured by the JBI score and sample size of study. There were no significant covariates associated with pooled cumulative survival (Table 2).

**Table 2:** Results of meta-regression analysis for pooled cumulative survival among patients who underwent prone positioning

Co-variate	No. studies	OR (95% CI)	*p* value
Pre-ECMO PF ratio	8	1.00 (95% CI 1.00–1.01)	0.28
Direct ARDS etiology	9	0.99 (95% CI 0.97–1.00)	0.15
Age	10	0.99 (95% CI 0.97–1.01)	0.37
Proportion of male	10	0.99 (95% CI 0.97–1.00)	0.12
Publication year	11	0.96 (95% CI 0.91–1.02)	0.21
JBI	11	1.02 (95% CI 0.85–1.21)	0.85
Sample size	11	1.00 (95% CI 0.99–1.01)	0.98

*OR* odds ratio, *95% CI* 95% confidence interval, *ARDS* Acute Respiratory Distress Syndrome, *ECMO* extracorporeal membrane oxygenation, *PF ratio* PaO_2_/FiO_2_ ratio, *JBI* Joanna Briggs Institute

### Secondary outcomes

ECMO duration was reported in 8 studies, and comparison with a control group was available in 6 studies. Patients undergoing PP (6 studies) had significantly longer ECMO duration, of an additional 9.6 days (Additional file 1: Fig. S2; 95% CI 5.5–13.7, *p* < 0.0001, very low certainty). Pooled ICU LOS (7 studies) was 42.5 days (95% CI 28.4–56.7, very low certainty). Comparing data from 3 studies reporting on ICU LOS, patients undergoing PP had significantly longer ICU stays by 14.5 days (Additional file 1: Fig. S3; 95% CI 3.4–25.7, *p* = 0.01) than control groups. There was no significant difference in survival to ECMO weaning between both groups of patients (3 studies; RR: 0.92, 95% CI 0.49–1.71, *p* = 0.78).

### ABG, ventilatory parameters and complications

Baseline and post-PP ABG values and ventilatory parameters were also compared as a proxy for evaluating the effect of PP on lung function, with the assumption of stable ECMO and sweep gas flows between the measures (summarized in Table 3). Due to the paucity of reported ABG and ventilatory parameters, particularly among matched cohorts, all available data were included in this analysis. A reported value was regarded as “Post-PP” if it was obtained at the end of a fixed-duration PP cycle or after patient has been returned to supine position. After PP, patients had significantly lower PaCO_2_ and driving pressure, as well as significantly improved PF ratios.

**Table 3:** Pooled baseline arterial blood gas and ventilator parameters with mean difference comparison

Parameter	Pooled estimate (95% CI)			*p* value
	Pre-PP	Post-PP	Mean difference	
PaO_2_	5 studies	5 studies	5 studies	0.10
	69.4 [63.3–75.4]	75.7 [65.2–86.3]	+ 5.1 [− 1.0 to + 11.2]	
PaCO_2_	6 studies	5 studies	5 studies	**0.03**
	44.7 [42.2–47.2]	43.7 [41.2–46.2]	− 1.5 [− 2.9 to − 0.2]	
pH	5 studies	4 studies	4 studies	0.98
	7.42 [7.41–7.43]	7.41 [7.40–7.43]	+ 0.0001 [− 0.01 to + 0.01]	
PF ratio	8 studies	7 studies	7 studies	**0.01**
	112.2 [92.2–132.3]	147.7 [131.4–164.0]	+ 24.9 [+ 6.5 to + 43.2]	
Tidal volume	4 studies	4 studies	4 studies	0.21
	3.2 [2.2–4.1]	3.7 [2.2–5.2]	+ 0.3 [− 0.2 to + 0.9]	
Respiratory rate	7 studies	6 studies	6 studies	0.08
	14.7 [13.0–16.4]	14.3 [11.8–16.7]	− 0.9 [− 2.0 to + 0.1]	
Driving pressure	3 studies	3 studies	3 studies	**0.01**
	11.5 [9.9–13.1]	10.7 [9.2–12.1]	− 0.8 [− 1.5 to − 0.2]	
Plateau pressure	5 studies	4 studies	4 studies	0.82
	26.2 [24.5–28.0]	27.1 [24.9–29.3]	− 0.1 [− 1.0 to + 0.8]	
PEEP	7 studies	6 studies	6 studies	0.88
	14.7 [13.8–15.6]	15.0 [13.7–16.4]	0.1 [− 0.9 to + 1.0]	
Sweep gas flow	7 studies	5 studies	5 studies	0.79
	6.0 [5.3–6.7]	6.5 [5.9–7.1]	+ 0.1 [− 0.3 to + 0.5]	
Respiratory system compliance	6 studies	6 studies	6 studies	0.12
	22.0 [20.5–23.6]	23.8 [21.3–26.3]	+ 1.8 [− 0.5 to + 4.0]	

*p*-value < 0.05 has been highlighted in bold

*PP* prone positioning, *PaO*_*2*_ partial pressure of oxygen, *PaCO*_*2*_ partial pressure of carbon dioxide, *PEEP* positive end expiratory pressure, *PF* PaO_2_/FiO_2_, *CI* confidence interval

A total of 533 ECMO complications and 24 PP complications were reported across 6 studies, which included 534 patients. The complications were grouped into broad categories, of which the most common complications reported were cardiovascular, hemorrhagic and mechanical complications. This trend was observed among both groups of patients. The most commonly reported complication related to PP is the development of low-grade pressure sores (12 of 24 PP complications). Major concerns like accidental decannulation, endotracheal tube displacement or accidental extubation were not reported in any of the studies. A summary of the complications reported is presented in Additional file 1: Table S6.

## Discussion

To our knowledge, this is the first systematic review and meta-analysis on the use of PP during ECMO in adult patients with ARDS. Patients who underwent PP during ECMO had a cumulative survival rate of 57%, less than the survival reported in the ECMO to Rescue Lung Injury in Severe ARDS (EOLIA) trial. PP during ECMO also resulted in improved oxygenation without an improvement in respiratory mechanics. However, PP resulted in an increased duration of ECMO run by 9.6 days and ICU LOS by 14.5 days and was comparable with some of the observational studies [21, 25]. Metaregression analysis identified no significant factors associated with survival for PP during ECMO.

PP has shown survival benefits in patients with ARDS by various mechanisms, however, the utility of this intervention during ECMO raises many questions. Franchineau et al. used electric impedance tomography (EIT) to describe the impact of PP in patients receiving VV ECMO and noted progressive redistribution of tidal volumes and end-expiratory lung impedance from ventral to dorsal regions with improvements seen in static lung compliance [17]. While we observed that gas exchange indices and driving pressure improved from PP during ECMO, other respiratory mechanics including compliance or plateau pressure did not change significantly despite the maneuver. However, given that few included studies comprehensively report these parameters, it may be difficult to draw reliable conclusions on the protective mechanism of PP. It is possible that an improved oxygenation may have resulted from an improved ventilation: perfusion matching upon PP [9].

Patients who undergo PP during ECMO have been shown to have significantly prolonged duration of ECMO support as well as ICU LOS [18, 19, 21, 24] It is likely that the patients subjected to PP during ECMO were sicker, which explains the need for the intervention as well as the longer duration of ECMO. Given the immortal time bias associated with interventions like ECMO, it is also plausible that sicker patients could have died earlier and thus did not undergo prone positioning, thereby accounting for the higher mortality. It is possible that PP, by alleviating VILI, may prevent ongoing native lung damage [28]. When this is associated with improvements in gas exchange, it may lead to improvements in right ventricular function and overall hemodynamics, thereby mitigating non-pulmonary organ failure. It was established that patients who underwent PP had greater extrapulmonary organ failure-free days up to 28 days after randomization in the PROSEVA trial [1]. It should also be noted that the control group mortality in some of the studies in our review that included a comparator arm was higher than reported in the treatment arm of the EOLIA trial and may account for the higher survival in patients with prone positioning seen in this analysis.

The survival rates reported in our analysis (57%) are less than that of EOLIA trial, which did not conduct PP in patients with ECMO. We also note that patients who were not treated with PP during VV ECMO had a lower pooled survival of 47% (95% CI 33.4–61.8), however, the calculated chances of survival between PP and control groups were statistically non-significant. Whether this is attributable to a greater severity of illness, or the variability in institutional workflows could not be ascertained from our review. In the absence of randomized controlled trials investigating the effect of PP with VV ECMO, it is difficult to conclusively determine if PP during VV ECMO has meaningful benefits. Nonetheless, the higher survival rates seen in patients undergoing PP during ECMO reported in this analysis are hypothesis generating for a future controlled trial. Routine PP of patients on VV ECMO and PP of patients with refractory hypoxia on VV ECMO represent two different clinical situations. Therefore, future controlled trials (ClinicalTrials.gov, ref. NCT04139733, NCT04607551) will have to test the risk to benefit ratio of routine PP during VV ECMO, focusing on patient centered outcomes. Prior to such a trial, standardization of data collection as well as patient management on VV ECMO, duration and timing of the PP sessions, as well as homogenizing the ECMO weaning criteria will be critical.

Strengths of this study include the broad inclusion criteria and relevant exclusion criteria, to ensure all relevant studies were included in the review. We reduced confounding by analyzing factors correlating with survival through additional subgroup analyses and metaregression. Single center data that overlapped were excluded thereby avoiding duplication of data. Nonetheless, we recognize several limitations of this study. Firstly, only 12 studies qualified for inclusion and all studies included were retrospective studies written in English. Certainly, meta-analyses of retrospective studies may be considered poorer evidence than randomized controlled trials, since they may be open to confounding factors as most included studies were not propensity score matched or risk adjusted. However, we note that our primary outcome was survival, for which data from retrospective studies have been viewed reliable and of high certainty when assessing with the GRADE method [29]. Mechanical ventilation was not standardized, adding in another layer of complexity. Meta-regression analyses are also inherently constrained by a lack of power, resulting in an increased risk of type 2 errors. We also recognize significant heterogeneity in survival outcomes reported, which may be due in part to the inclusion of studies with COVID-19 patients who often have differing clinical presentation than other causes of ARDS. It is also notable that some of the studies had reported much lower survival (14%, 21%) than the pooled average (57%) [18, 26], possibly from inclusion of an older and sicker cohort of patients. We also note that included studies have varying PP strategies and protocols (eg. physician discretion, refractory hypoxemia, number of days on mechanical ventilation) which also likely has contributed to the heterogeneity observed. Given the heterogeneity and small number of studies reporting on specific outcomes (eg. survival to ECMO weaning), these results should be interpreted with caution. Additionally, there was no significant publication bias in the studies included and JBI critical appraisal deemed most of the articles as high quality and suitable for inclusion while the GRADE assessment suggested a high certainty of evidence for the primary outcome.

## Conclusion

Prone positioning during ECMO for ARDS has a cumulative survival of 57% suggesting that patients undergoing PP during ECMO may have clinically meaningful benefits. However, the heterogeneity of outcomes in included studies may indicate that the treatment effects of PP on VV ECMO may vary between patients. Furthermore, the intervention leads to longer ECMO runs and ICU LOS. To what extent combining PP during ECMO extends ECMO duration and ICU length of stay warrants further investigation. The timing of PP during ECMO as well as the duration of PP needed to achieve a meaningful clinical benefit is currently unclear. Future studies should identify the patients who are likely to benefit most from PP before adoption of PP routinely in select patients undergoing ECMO for ARDS.

## Supplementary Information

**Additional file 1.** Supplementary tables and figures.

## Data Availability

Data sharing is not applicable to this article as no datasets were generated or analysed during the current study. All data analysed in this study are included in the published studies and their supplementary information files.
