# Supplementary material for: Prone positioning during venovenous extracorporeal membrane oxygenation for acute respiratory distress syndrome: a systematic review and meta-analysis
Source: Crit Care. 2021 Aug 12;25:292. doi: 10.1186/s13054-021-03723-1 (PMC8358249; doi:10.1186/s13054-021-03723-1)
Supplement: Supplementary file 1 — Additional file 1. Supplementary tables and figures. [file 13054_2021_3723_MOESM1_ESM.docx]

**PRONE POSITIONING DURING VENOVENOUS EXTRA CORPOREAL MEMBRANE OXYGENATION FOR ACUTE RESPIRATORY DISTRESS SYNDROME: A SYSTEMATIC REVIEW AND METANALYSIS**

**ADDITIONAL FILE 1: SUPPLEMENTARY MATERIAL**

**Wynne Hsing Poon*^1^, MBBS, (ORCID: 0000-0003-3320-8038)**

**Kollengode Ramanathan*^1,2,8^, MD, FCICM, (ORCID: 0000-0003-1822-9455)**

**Ryan Ruiyang Ling^1^, MBBS, (ORCID: 0000-0002-8335-7640)**

**Isabelle Xiaorui Yang^1^, MBBS (ORCID: 0000-0002-9064-4218)**

**Chuen Seng Tan^3^, PhD, (ORCID: 0000-0002-6513-2309)**

**Matthieu Schmidt^4,5^, MD, PhD, (ORCID: 0000-0002-2931-4412)**

**Kiran Shekar^6,7,8^, FCICM, PhD, (0000-0002-1239-7514)**

***Both authors contributed equally to the manuscript.**

^1^ Yong Loo Lin School of Medicine, National University of Singapore, Singapore.

^2^ Cardiothoracic Intensive Care Unit, National University Heart Centre, National University Hospital, Singapore

^3^ Saw Swee Hock School of Public Health, National University of Singapore, Singapore.

^4^ Service de Médecine Intensive-Réanimation, Institut de Cardiologie, Assistance Publique-Hôpitaux de Paris, Hôpital Pitié-Salpêtrière, Paris, France

^5^ Sorbonne Université, GRC 30, Reanimation et Soins intensifs du Patient en Insuffisance Respiratoire aigüE, AP-HP, Hôpital de la Pitié Salpêtrière, F-75013, Paris, France.

^6^ Adult Intensive Care Services, Prince Charles Hospital, Brisbane, Queensland, Australia

^7^ Queensland University of Technology, Brisbane; University of Queensland, Brisbane

^8^ Bond University, Gold Coast, Queensland, Australia

**Corresponding author:**

**Kollengode Ramanathan,**

**Cardiothoracic Intensive Care Unit, National University Heart Centre, National University Hospital, Singapore. Level 9, 1E Kent Ridge Road, SINGAPORE 119228.**

**Tel: +6567727862**

**Email:** [**ram_ramanathan@nuhs.edu.sg**](mailto:ram_ramanathan@nuhs.edu.sg)**.**

**Supplementary Table 1.** Search strategy for the systematic review and meta-analysis

| PubMed | |
| --- | --- |
| 1 | "Extracorporeal Membrane Oxygenation"[Mesh] |
| 2 | (ECMO[Title/Abstract] OR "Extracorporeal Membrane Oxygenat*"[Title/Abstract] OR "Extracorporeal Life Support*"[Title/Abstract] OR ECLS[Title/Abstract] OR "membrane oxygenat*"[Title/Abstract]) |
| **3** | #1 OR #2  (ECMO[Title/Abstract] OR "Extracorporeal Membrane Oxygenat*"[Title/Abstract] OR "Extracorporeal Life Support*"[Title/Abstract] OR ECLS[Title/Abstract] OR "membrane oxygenat*"[Title/Abstract]) OR ("Extracorporeal Membrane Oxygenation"[Mesh]) |
| 4 | "Prone Position"[Mesh] |
| 5 | (“prone position*”[Title/Abstract] OR pron*[Title/Abstract] OR “positioning therapy”[Title/Abstract]) |
| 6 | (“Prone position”[Mesh]) OR (“prone position*”[Title/Abstract] OR pron*[Title/Abstract] OR “positioning therapy”[Title/Abstract]) |
| 7 | 3 and 6  ("Prone position"[MeSH Terms] OR ("prone position*"[Title/Abstract] OR "pron*"[Title/Abstract])) AND ((((("ECMO"[Title/Abstract] OR "extracorporeal membrane oxygenat*"[Title/Abstract]) OR "extracorporeal life support*"[Title/Abstract]) OR "ECLS"[Title/Abstract]) OR "membrane oxygenat*"[Title/Abstract]) OR "Extracorporeal Membrane Oxygenation"[MeSH Terms]) |
| Cochrane | |
| 1 | MeSH descriptor: [Extracorporeal Membrane Oxygenation] explode all trees |
| 2 | (ECMO OR “Extracorporeal membrane oxygenat*” OR “Extracorporeal Life Support*” OR ECLS OR “membrane oxygenat*”):ti,ab,kw |
| 3 | MeSH descriptor: [Prone Position] explode all trees |
| 4 | (“prone position*” OR pron* OR “positioning therapy”):ti,ab,kw |
| 5 | (#1 OR #2) AND (#3 OR #4) |
| Embase | |
| 1 | 'extracorporeal oxygenation'/exp OR 'ecmo':ab,kw,ti OR 'ecls':ab,kw,ti OR 'extracorporeal membrane oxygenat*':ab,kw,ti OR 'extracorporeal life support*':ab,kw,ti OR 'membrane oxygenat*':ab,kw,ti |
| 2 | 'prone position'/exp OR 'prone position*':ab,ti,kw OR 'pron*':ab,ti,kw OR ‘positioning therapy’:ab,ti,kw |
| 3 | #1 AND #2 |
| 4 | #1 AND #2 AND [english]/lim AND [article]/lim |
| Scopus | |
| 1 | TITLE-ABS-KEY(“Extracorporeal membrane oxygenat*” OR “ECMO” OR “ECLS” OR “Extracorporeal life support*” OR “membrane oxygenat*”) |
| 2 | TITLE-ABS-KEY(“pron* position*” OR “pron*” OR “positioning therapy”) |
| 3 | #1 AND #2 |
| 4 | #3 lim:english, Lim: article |

**Supplementary Figure 1**. PRISMA flowchart for the systematic review and meta-analysis


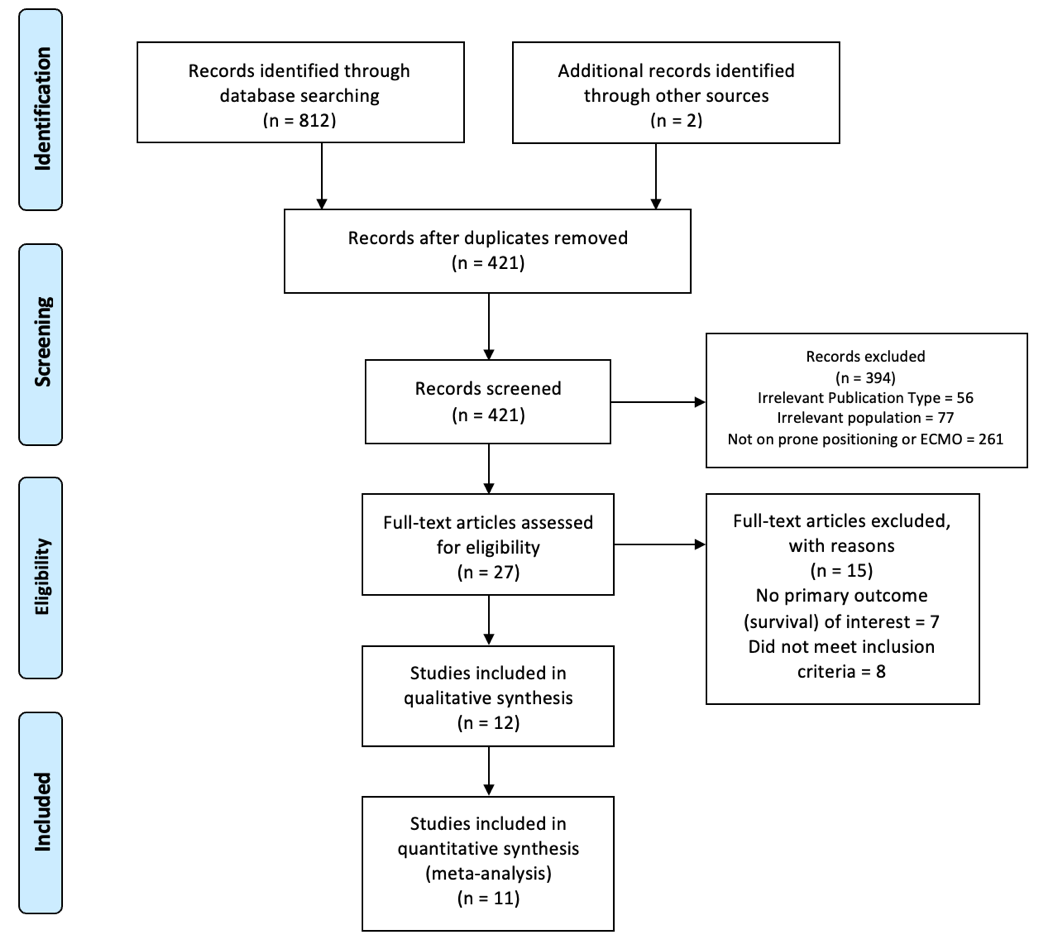


**Supplementary Figure 2.** Forest plot for mean difference in ECMO duration.


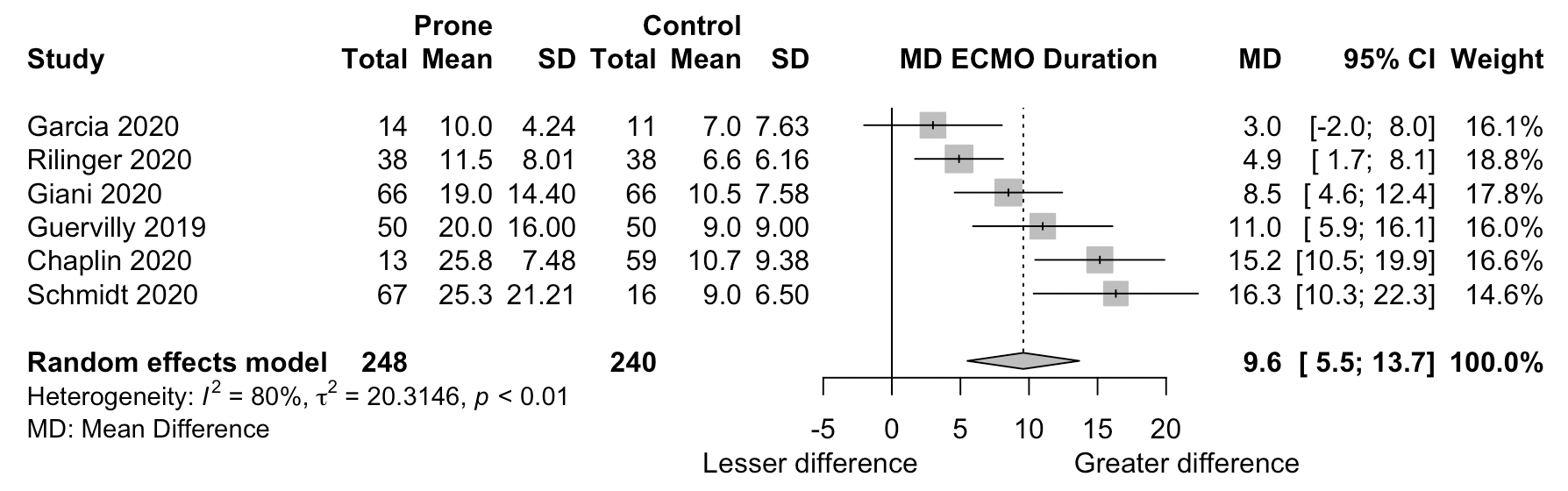


**Supplementary Figure 3.** Forest plot for mean difference in ICU LOS.


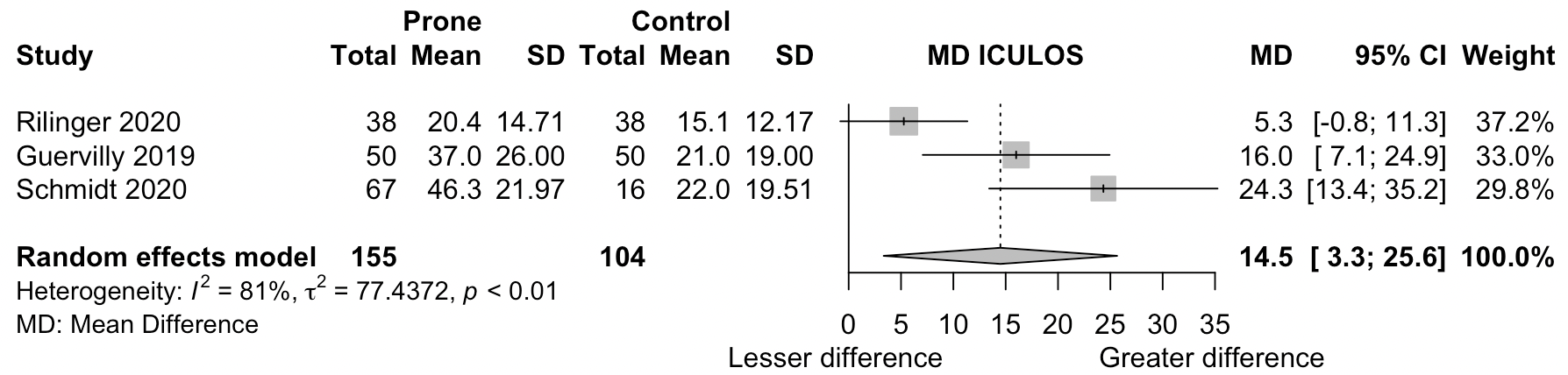


**Supplementary Table 2:** Summary of review of literature on prone positioning of patients during ECMO

| **Author** | **Country** | **Total number of patients(n)** | **Proned** | | | **Control** | | | **Survival definition** | **Baseline PF of Proned** | **Indications for Prone Positioning** | **Proning Regime** | **Duration of Proning** |
| --- | --- | --- | --- | --- | --- | --- | --- | --- | --- | --- | --- | --- | --- |
|  |  |  | **Patients**  **(n)** | **Survived (n)** | **ECMO Duration(days)** | **Patients**  **(n)** | **Survived (n)** | **ECMO Duration(days)** |  |  |  |  |  |
| Kipping 2013 | Germany | 12 | 12 | 6 | 10.6 ± 7.7 |  |  |  | Discharge | 49.7±31.0 | Severely hypoxemic ARDS-patients while on ECMO  *it was an inclusion criteria and hence not elaborated* | Fixed | 8h/session |
| Guervilly 2014 | France | 15 | 15 | 8 | NR |  |  |  | ICU | 105.3±46.6 | One of the three following conditions:  1) severe hypoxemia (PaO2/FiO2 ratio <70) despite FDO2 and FiO2 both at 100%,  2) injurious ventilation parameters with plateau pressure exceeding 32 cmH2O despite the reduction of tidal volume to 2-3 mL/kg and the reduction of PEEP to 10 cmH2O  3) failure of attempt to wean ECMO after at least 10 days of ECMO and the presence of lung consolidations on chest ultrasonography. | Not fixed | Median 1 session/patient of median duration 12h |
| Kimmoun 2015 | France | 17 | 17 | 16 | 19 ± 10.5 |  |  |  | Discharge | 107.7±35.6 | One of the two following conditions:  (1) Failure of attempts to wean VV-ECMO after at least 7 days under VV-ECMO combined with the need of therapeutic sedation  (2) Refractory hypoxemia with PaO2/FiO2 ratio <85 mmHg under FiO2 100 % both on the ventilator and the mem- brane despite optimal VV-ECMO and ventilator settings combined or not with persistent high plateau pressure (>25 cmH2O) despite ultra-protective ventilation. | Fixed | 24h/session |
| Lucchini 2018 | Italy | 14 | 14 | 9 | 35.7 ± 31.3 |  |  |  | Discharge | 113.3±43.7 | ARDS patients on VV-ECMO  *It was an inclusion criteria and hence not elaborated* | Not fixed | 9.4±4.7h/session |
| Guervilly 2019* | France | 100 | 50 | 30-day: 38 60-day: 34 90-day: 32 | 20 ± 14 | 50 | 30-day: 25 60-day: 23 90-day: 21 | 9 ± 9 | 30-day/60-day/90-day | 67 ± 21 | Patients were considered for PP in the cases of:  1) persistent hypoxemia defined by SpO2 <88 % or PaO2 < 55 mmHg despite 100% FdO2 and FiO2 with a maximal ECMO blood flow  2) failure of attempt to wean ECMO after at least 10 days of ECMO and the presence of lung consolidations on chest X-ray or lung ultrasounds  3) according to the physician in charge of the patient. | Fixed | 12-16 h/session |
| Chaplin 2020 | New Zealand | 72 | 13 | 9 | 25.8 ± 7.5 | 59 | 41 | 10.7 ± 9.4 | 6 months | 143.3±25.7 | Patients who received VV-ECMO for respiratory failure  *It was an inclusion criteria and hence not elaborated* | Not fixed | 2.0±1.5 sessions for 11.4±7.2h |
| Franchineau 2020 | France | 21 | 21 | 13 | 16.7±9.5 |  |  |  | ICU |  | ECMO-supported severe ARDS patients  *it was an inclusion criteria and hence not elaborated* | Fixed | 16h/session 1.7±0.8 sessions conducted/patient |
| Garcia 2020 | France | 25 | 14 | ICU: 2^^^  28-day: 3 | 10 ± 4.2 | 11 | ICU: 8  28-day: 8 | 7 ± 7.6 | ICU/28-day^§^ | 82.3±22.5 | PP was considered in case of severe hypoxemia (PaO2/FiO2 ratio below 80 mmHg) despite FDO2 and FiO2 both at 100% and in case of extensive lung consolidation (ECL) on chest imaging (>50% of lung volume) for patients with ARDS on VV-ECMO | Not fixed | Min. 1x/day Mean duration/session: 16±1.7h |
| Giani 2020* | Italy | 132 | 66 | 46 | 19 ± 14.3 | 66 | 35 | 11.3 ± 9.0 | Discharge | 73±29 | Diagnosis of ARDS according to Berlin definition and treated with veno-venous ECMO, where proning is routinely performed during extracorporeal support | Not fixed | 15±4.51h/session |
| Rilinger 2020* | Germany | 76 | 38 | ICU: 14  Discharge: 14  30-day: 18 | 11.5 ± 8.0 | 38 | ICU: 14  Discharge: 14  30-day: 16 | 5.9 ± 5.0 | ICU/Discharge/ 30-day | 81.0±34.7 | All patients had severe ARDS, decision to perform proning in the individual case lays with the treating medical team’s judgement. | Not fixed | 19.0±3.1h /session mean 2±1.5 sessions/patient |
| Schmidt 2020 | France | 83 | 67 | 50 | 25.3 ± 21.2 | 16 | 8 | 9 ± 6.5 | 60-day |  | Patients with confirmed SARS-CoV-2 infection and treated with ECMO received venoarterial-ECMO or venovenous-ECMO for severe ARDS. Early prone-positioning on ECMO was encouraged in the absence of haemodynamic instability and contraindications for prone-positioning (ie, massive haemoptysis requiring an immediate surgical or interventional radiology procedure; deep venous thrombosis treated for less than 2 days, or single anterior chest tube with air leaks) | Fixed | 16h/session |
| Yang 2021 | China | 73 | 51 | 11 |  | 22 | 3 |  | Discharge |  | Patients infected by SARS-CoV-2 who were treated with ECMO |  |  |

**Propensity-score matched/Risk-adjusted data were used in the meta-analysis of primary outcome for these studies and is reflected similarly in this table.*

*^As fewer patients survived to ICU discharge than at 28 days in this study, survival to ICU discharge was regarded as the longer time interval and used in meta-analysis of cumulative survival.*

*§ 28 day survival for this study was rounded off and pooled with other available data for a summary of 30 day survival*

*(VV-)ECMO: (Veno-Venous) Extracorporeal Membrane Oxygenation, PF: PaO2:FiO2 ratio, ICU: Intensive Care Unit, ARDS: Acute Respiratory Distress Syndrome, SARS-CoV2: Severe Acute Respiratory Syndrome Coronavirus 2*

**Supplementary Table 3**. Summary of JBI Scores for Prevalence Studies of included studies

| **Study/Qn** | **1** | **2** | **3** | **4** | **5** | **6** | **7** | **8** | **9** | **Total** |
| --- | --- | --- | --- | --- | --- | --- | --- | --- | --- | --- |
| Franchineau 2020 | **✓** | **✓** | **✓** | **✓** | **✓** | 0 | 0 | **✓** | **✓** | 7 |
| Garcia 2020 | **✓** | **✓** | **✓** | **✓** | **✓** | **✓** | **✓** | **✓** | **✓** | 9 |
| Kimmoun 2015 | **✓** | **✓** | **✓** | **✓** | **✓** | **✓** | **✓** | **✓** | **✓** | 9 |
| Kipping 2013 | **✓** | **✓** | **✓** | **✓** | **✓** | 0 | 0 | **✓** | **✓** | 7 |
| Lucchini 2018 | **✓** | **✓** | **✓** | **✓** | **✓** | **✓** | **✓** | **✓** | **✓** | 9 |
| Rilinger 2020 | **✓** | **✓** | **✓** | **✓** | **✓** | **✓** | **✓** | **✓** | **✓** | 9 |
| Giani 2020 | **✓** | **✓** | **✓** | **✓** | **✓** | **✓** | **✓** | **✓** | **✓** | 9 |
| Guervilly 2019 | **✓** | **✓** | **✓** | **✓** | **✓** | 0 | **✓** | **✓** | **✓** | 8 |
| Chaplin 2020 | **✓** | **✓** | **✓** | **✓** | **✓** | 0 | 0 | **✓** | **✓** | 7 |
| Guervilly 2014 | **✓** | **✓** | **✓** | **✓** | **✓** | 0 | **✓** | **✓** | **✓** | 8 |
| Schmidt 2020 | **✓** | **✓** | **✓** | **✓** | **✓** | **✓** | **✓** | **✓** | **✓** | 9 |
| Yang 2021 | **✓** | **✓** | **✓** | **✓** | **✓** | **✓** | 0 | **✓** | **✓** | 8 |

**Supplementary Table 4.** Grading of Recommendations Assessments, Developments and Evaluations (GRADE) approach for assessing certainty of evidence of study outcomes for pooled cumulative survival

| **№ of studies** | **Certainty assessment** | | | | | | **Effect** | | | **Certainty** | **Importance** |
| --- | --- | --- | --- | --- | --- | --- | --- | --- | --- | --- | --- |
|  | **Study design** | **Risk of bias** | **Inconsistency** | **Indirectness** | **Imprecision** | **Other considerations** | **№ of events** | **№ of individuals** | **Rate (95% CI)** |  |  |
| Cumulative Survival | | | | | | | | | | | |
| 11 | observational studies | not serious | not serious ^a^ | not serious | not serious ^b^ | none | - | 363 | mean 56.93 % (41.89 - 71.38) | ⨁⨁⨁⨁ HIGH | CRITICAL |

#### Explanations

a. There was significant heterogeneity I2 = 58%. Apart from one study, most of the confidence intervals overlap and point estimates were reasonably close. Hence this was a borderline decision to not rate down for inconsistency.

b. While the 95%CI is relatively wide to the pooled estimate, and sample size is small, the pooled survival is comparable to the ELSO International Summary 2020 for VVECMO, the decision to initiate prone positioning should not change at either ends of the CI.

**Supplementary Table 5.** Grading of Recommendations Assessments, Developments and Evaluations (GRADE) approach for assessing certainty of evidence of study outcomes for comparison of cumulative survival, ECMO duration and ICU LOS

| **Certainty assessment** | | | | | | | **№ of patients** | | **Effect** | | **Certainty** | **Importance** |
| --- | --- | --- | --- | --- | --- | --- | --- | --- | --- | --- | --- | --- |
| **№ of studies** | **Study design** | **Risk of bias** | **Inconsistency** | **Indirectness** | **Imprecision** | **Other considerations** | **Prone positioning** | **Supine positioning** | **Relative (95% CI)** | **Absolute (95% CI)** |  |  |
| **Cumulative Survival** | | | | | | | | | | | | |
| 7 | observational studies | not serious | not serious | not serious | serious ^a^ | none | 208/299 (61.6%) | 130/262 (48.0%) | **RR 1.19** (0.92 to 1.55) | **94 more per 1,000** (from 40 more to 273 more) | ⨁◯◯◯ VERY LOW | CRITICAL |
| **ICU LOS** | | | | | | | | | | | | |
| 3 | observational studies | not serious | serious ^b^ | not serious | not serious | none | 155 | 104 | - | MD 14.50 **days more** (3.35 more to 25.65 more) | ⨁◯◯◯ VERY LOW | IMPORTANT |
| **ECMO Duration** | | | | | | | | | | | | |
| 6 | observational studies | not serious | serious ^b^ | not serious | not serious | none | 248 | 240 | - | MD 9.59 **days more** (5.51 more to 13.67 more) | ⨁◯◯◯ VERY LOW | IMPORTANT |

**CI:** Confidence interval; **RR:** Risk ratio; **MD:** Mean difference

#### Explanations

a. The sample size is relatively small and there is a wide 95%CI which overlaps no effect (1). As such, this was rated down for imprecision.

b. There was considerable variability in point estimates, with notable marginal CI overlap in one of 3 studies. As such, this was rated down for inconsistency.

**Supplementary Table 6:** Summary of ECMO and PP related complications reported

| **Study*** |  | **ECMO Complications** | | | | | | | | | **PP Complications** | | | |
| --- | --- | --- | --- | --- | --- | --- | --- | --- | --- | --- | --- | --- | --- | --- |
|  | **PP groups** | | | | | | | | | | | | | |
|  | No. of Patients | Cardiovascular | Haemorrhagic | Mechanical | Respiratory | Renal | Neurological | Infectious | Metabolic | Other | Pressure sores | Drop in ECMO Flow | Swelling | Tubes dislodged |
| Kimmoun 2015 | 17 | 1 |  |  |  |  |  |  |  |  |  | 1 |  | 0 |
| Kipping 2013 | 12 | 4 | 39 |  | 10 |  |  |  |  | 1 |  | 1 |  | 1 |
| Guervilly 2019 | 91 | 111 | 50 | 46 |  |  |  |  |  |  |  |  |  |  |
| Chaplin 2020 | 13 | 2 | 2 | 2 | 2 | 4 |  |  | 1 | 1 | 6 |  |  | 0 |
| Garcia 2020 | 14 |  | 3 |  | 2 |  |  |  |  |  | 6 | 3 |  |  |
| Giani 2020 | 107 | 2 | 4 |  | 9 |  |  |  |  | 1 |  | 4 | 2 |  |
| Total PP Complications | | 120 | 98 | 48 | 23 | 4 | 0 | 0 | 1 | 3 | 12 | 9 | 2 | 1 |
|  | **Control groups** | | | | | | | | | | | | | |
| Guervilly 2019 | 77 | 86 | 32 | 32 |  |  |  |  |  |  |  | | | |
| Giani 2020 | 133 | 6 | 4 |  |  |  |  |  |  |  |  |  |  |  |
| Chaplin 2020 | 59 | 9 | 14 | 14 | 1 | 22 | 6 | 1 | 4 | 5 |  |  |  |  |
| Total Control Complications: | | 101 | 50 | 46 | 1 | 22 | 6 | 1 | 4 | 5 |  |  |  |  |

**Studies that did not report complications were not included.*

*ECMO: Extracorporeal Membrane Oxygenation, PP: Prone positioning*
